# Integrative analysis of common genes and driver mutations implicated in hormone stimulation for four cancers in women

**DOI:** 10.7717/peerj.6872

**Published:** 2019-06-06

**Authors:** Salma Begum Bhyan, YongKiat Wee, Yining Liu, Scott Cummins, Min Zhao

**Affiliations:** 1 Faculty of Science, Health, Education and Engineering, University of the Sunshine Coast, Sunshine Coast, QLD, Australia; 2 The School of Public Health, Institute for Chemical Carcinogenesis, Guangzhou Medical University, Guangzhou, China

**Keywords:** Cancer genomics, Network analysis, Gynaecological cancer, Endometrial cancer, Ovarian cancer, Breast cancer, Cervical cancers oncogene, Tumour suppressor gene

## Abstract

Cancer is one of the leading cause of death of women worldwide, and breast, ovarian, endometrial and cervical cancers contribute significantly to this every year. Developing early genetic-based diagnostic tools may be an effective approach to increase the chances of survival and provide more treatment opportunities. However, the current cancer genetic studies are mainly conducted independently and, hence lack of common driver genes involved in cancers in women. To explore the potential common molecular mechanism, we integrated four comprehensive literature-based databases to explore the shared implicated genetic effects. Using a total of 460 endometrial, 2,068 ovarian, 2,308 breast and 537 cervical cancer-implicated genes, we identified 52 genes which are common in all four types of cancers in women. Furthermore, we defined their potential functional role in endogenous hormonal regulation pathways within the context of four cancers in women. For example, these genes are strongly associated with hormonal stimulation, which may facilitate rapid diagnosis and treatment management decision making. Additional mutational analyses on combined the cancer genome atlas datasets consisting of 5,919 gynaecological and breast tumor samples were conducted to identify the frequently mutated genes across cancer types. For those common implicated genes for hormonal stimulants, we found that three quarter of 5,919 samples had genomic alteration with the highest frequency in *MYC* (22%), followed by *NDRG1* (19%), *ERBB2* (14%), *PTEN* (13%), *PTGS2* (13%) and *CDH1* (11%). We also identified 38 hormone related genes, eight of which are associated with the ovulation cycle. Further systems biology approach of the shared genes identified 20 novel genes, of which 12 were involved in the hormone regulation in these four cancers in women. Identification of common driver genes for hormone stimulation provided an unique angle of involving the potential of the hormone stimulants-related genes for cancer diagnosis and prognosis.

## Introduction

Cancer is one of the leading causes of death of women worldwide (Ferlay et al., 2015; Ginsburg et al., 2017). Breast, ovarian, endometrial and cervical cancers are the four common cancers of women and, in addition to the distress caused to the sufferer, there is a considerable burden on the public health services (Torre et al., 2017). Globally, more than five million women died in 2012 as a result of breast cancer. Besides, cervical, ovarian and endometrial cancers were the cause of death of 2,66,000, 1,52,000 and 76,000 women, respectively (Ferlay et al., 2015). Every year >2 million women are diagnosed with breast or cervical cancer, although a large number remain undiagnosed (Ferlay et al., 2015). Population growth along with the increase of life expectancy these numbers are scaling up worldwide (Chen et al., 2016). According to cancer statistics 2018 (Siegel, Miller & Jemal, 2018), more than 30% of the projected new cancer cases in women in the US are breast cancer, 1% cervical, 7% endometrial and 2.5% ovarian cancers. Development of appropriate diagnostic tools and early screening can improve effective treatment and the chances of survival. For example, for 90% of breast cancer cases, women have an increased chance of survival for at least five more years, if diagnosed at an early stage of cancer development (American Chemical Society, 2018). Likewise, in ovarian cancer, 5-year survival rates can increase from 5% to 90% with early detection (American Chemical Society, 2018). The reason behind this is that in the initial stages the cancer cells are confined to a small area and in a small number of cells, hence offers the best chance for effective treatment (Smith et al., 2003). Developing early detection tools may increase treatment options and result in an improved quality of life and survival rates for patients.

Identification of common driver genes facilitates rapid cross-cancer diagnosis in the early stages of carcinogenesis. Based on the effect on cancer progression, two groups of driver genes are defined: oncogenes (OGs) and tumor-suppressor genes (TSGs). OGs are mutated forms of proto-oncogenes, which produce proteins that enhance cell division or inhibit apoptosis (Hartwell et al., 1999). Usually, OGs are dominant mutations, and even a single copy mutation can play a part in tumor formation (Does, Thiel & Johnson, 2003). On the other hand, TSGs may slow down cell growth by producing proteins that inhibit cell division, repair DNA errors and regulate apoptosis (Does, Thiel & Johnson, 2003). In general, mutations in TSGs are usually recessive and show a loss of activity only when both copies of the genes are mutated (Sager, 1989). Although a substantial number of OG and TSG driver mutations have been identified, only a few studies have been undertaken to explore their significance in cancer diagnosis (Futreal et al., 2004).

To date, the pathogenesis of cancers in women was not clearly defined, and therefore, comprehensive diagnosis and proper treatments are still unavailable. Unlike other Mendelian disorders, cancer is mainly driven by multiple genes causing somatic genetic variation. These multiple mutations act together on particular somatic cell populations to proliferate the cells efficiently than their neighbor cells (Alexandrov et al., 2013; Stratton & Rahman, 2008). Inherent multidimensional genomic complexity causes different types of somatic genetic variations including single nucleotide variants, short insertion and deletion, large copy number alterations, and structural rearrangements (Ciriello et al., 2013). Recent advancement of in high-throughput sequencing technologies generated a large number of data on these genetic variants in human (Capriotti et al., 2012; The 1000 Genomes Project Consortium, 2012) and allows us to find out the relationship between the phenotypic expression and the genes associated with genetic disorders (Bamshad et al., 2011) like cancers in women.

In 2005, the cancer genome atlas (TCGA) project was commenced to create a comprehensive public genomic profile of sequenced data on more than 30 cancer types (Tomczak, Czerwińska & Wiznerowicz, 2015). These data provided an open-access platform for functional cancer genomics studies to identify the essential genes and their regulatory networks. Over the last decade, TCGA has been widely used to explore genomic and pan-genomic studies. Recently a pan-genomic study has been undertaken to identify both similarities and differences among different type of gynaecologic cancers and non-gynaecologic breast cancers. Using TCGA samples from 2,579 patients, Berger et al. (2018) identified shared characteristics and unique molecular features of the tumors, and classified patient samples into prognostic molecular subtypes to suggest potential therapeutic targets. Till today, a large number of small scale research has been undertaken on different types of cancers. Initiatives have also been taken to integrate all the literature-based evidence in separate databases. ECGene (Zhao, Liu & O’mara, 2016), OCGene (Liu et al., 2015), G2SBC (Mosca et al., 2010) and CCDB (Agarwal et al., 2010) are the four literature-based databases providing all available literature evidence on endometrial, ovarian, breast and cervical cancers, respectively. Mining these four databases can be instrumental in exploring the critical genes involved in all these four cancers in women.

Hormones play functional roles in the etiology of breast, endometrial and ovarian cancers in women (Chen, Brown & Yager, 2008). There is evidence of an association between circulating hormones and cancers in women. The sex steroid hormones including estrogen and testosterone are associated with an increased risk of breast cancer (Collaborative Group on Hormonal Factors in Breast Cancer, 1997; Endogenous Hormones and Breast Cancer Collaborative Group, 2013). It is now established that endogenous hormones play a role in premenopausal breast cancer (Endogenous Hormones and Breast Cancer Collaborative Group, 2013). However, evidence of hormonal associations for endometrial, ovarian and cervical cancers is limited. The estrogen-mediated mitochondrial pathway in breast cancer is well understood (Pedram et al., 2006), but the regulatory pathway remains unclear for the other three common cancers in women. Identification of common genes functioning in endogenous hormone-regulated carcinogenesis and the exploration of the pathways can help to take preventive measures against the risk factors associated with specific cancers. In this study, we aimed to (i) identify common genes implicated as cancer and cancer driver genes for four cancers in women; (ii) discover the mutational and functional effects of common driver genes; and (iii) explore driver genes involved in endogenous hormonal regulation pathways in four cancers in women.

## Materials and Methods

### Extraction of a gene list implicated in four cancers in women

To understand the genes implicated in four cancers in women, we downloaded the gene lists from the four literature-based databases. Our selection criteria for the dataset included literature-based curation and comprehensiveness of annotated genes (>500 genes). The four databases were:

- ECGene (Zhao, Liu & O’mara, 2016): a literature-based collection of endometrial cancer genes, which generated and comprehensively annotated a list of 458 EC‐implicated genes from 824 PubMed abstracts, including 423 protein-coding and 35 non-coding human genes, and 360 co-expressed lncRNAs with 357 endometrial cancer genes.
- OCGene (Liu et al., 2015): an online database for in-depth analysis of ovarian cancer genes. The database contains 2,068 manually curated experimentally verified human genes from 2,825 PubMed abstracts and precomputed regulatory motifs involved in transcription factors, microRNAs and long non-coding RNAs.
- G2SBC (Mosca et al., 2010): a genes-to-systems breast cancer database which integrates published data of altered genes, transcripts and proteins associated with breast cancer cells. Additionally, G2SBC provides an ontology-based query system and analysis tools related to intracellular pathways, network analysis of protein-protein interactions, protein structure and systems modeling, all enabling the study of breast cancer using a multilevel perspective.
- CCDB (Agarwal et al., 2010): a cervical cancer gene database, which is a manually curated catalogue of 537 experimentally validated genes involved in the different stages of cervical cancer.

A Venn diagram was plotted using an online analysis tool (http://bioinformatics.psb.ugent.be/webtools/Venn/) to identify the overlapping of cancer implicated genes (Table S1). Venn diagram analysis also provided a list of common cancer implicated genes involved in different type of cancers in women.

### Mutational analysis

To assess the mutational profile of the genes that were common to the four cancers, we conducted oncoprint and mutation tests using the cBioportal website (Cerami et al., 2012). The cBioportal analysis helps in exploring, visualizing, and analyzing multidimensional cancer genomics data, which can be summarized as genomic alterations. Mutational analysis provides the frequency of mutation and mutual exclusivity of the common genes used in this study. We used Fisher’s exact test to determine *P*-values using the null hypothesis that there is a proportional relationship in the frequency of alteration of two genes. Visualization of mutation frequency can be exploited to identify the degree of penetrance of the gene in the database samples. The mutual exclusivity analysis was used to determine the potential complementary mechanism for some gene pairs contributing to oncogenesis and cancer progression.

### Functional enrichment analysis

To assess the molecular functions of the common genes identified, we conducted functional enrichment tests using Toppfun (Chen et al., 2009), a web-based database tool. The molecular function of gene ontology (GO), cellular components, biological process, and pathways-based enrichment analyses were examined to provide an updated collection of genome annotations. We considered the GO IDs and their corresponding *P*-values for the visualization process using REVIGO (Supek et al., 2011), which removed the redundant GO terms. The GO results served as the input data in REVIGO and subsequently produced semantic similarity-based scatterplots of GO terms from Toppfun.

### Biological network integration and novel gene identification

Results of Toppfun (Chen et al., 2009) analysis was used as an important tool for interpreting the growing amount of biological data from genomic studies, which captures knowledge of biological processes at the molecular level. To present the networks and to categorize the functions of the networks, we utilized the network topological properties (Assenov et al., 2007) (e.g., degree and shortest path), which were calculated from the network. The degree of a node (*k*) indicates the number of links connected to the node and shows the biological relevance of the node. A high *k* value indicates that the node has a central regulatory role. The shortest path (*d*) is the shortest transverse distance between two nodes and indicates the probability of a protein being functionally relevant for other proteins. We deployed the network analyzer plugin (Lopes et al., 2010; Shannon et al., 2003) to compute the topological properties in the oncogene network. The network layout was constructed based on Cytoscape v3.6 (Shannon et al., 2003). We further analyzed the common driver gene list using GeneMANIA (Warde-Farley et al., 2010), which provides biological information including protein and gene interactions, co-expression, co-localization, protein domains and physical interactions. Gene networks show the relation between the common genes and may help to identify novel genes, which are not included in the driver gene list but play significant role in the oncogenesis of the four cancers in women.

## Results

### Identification and mutational analysis of common genes

**Figure 1:**
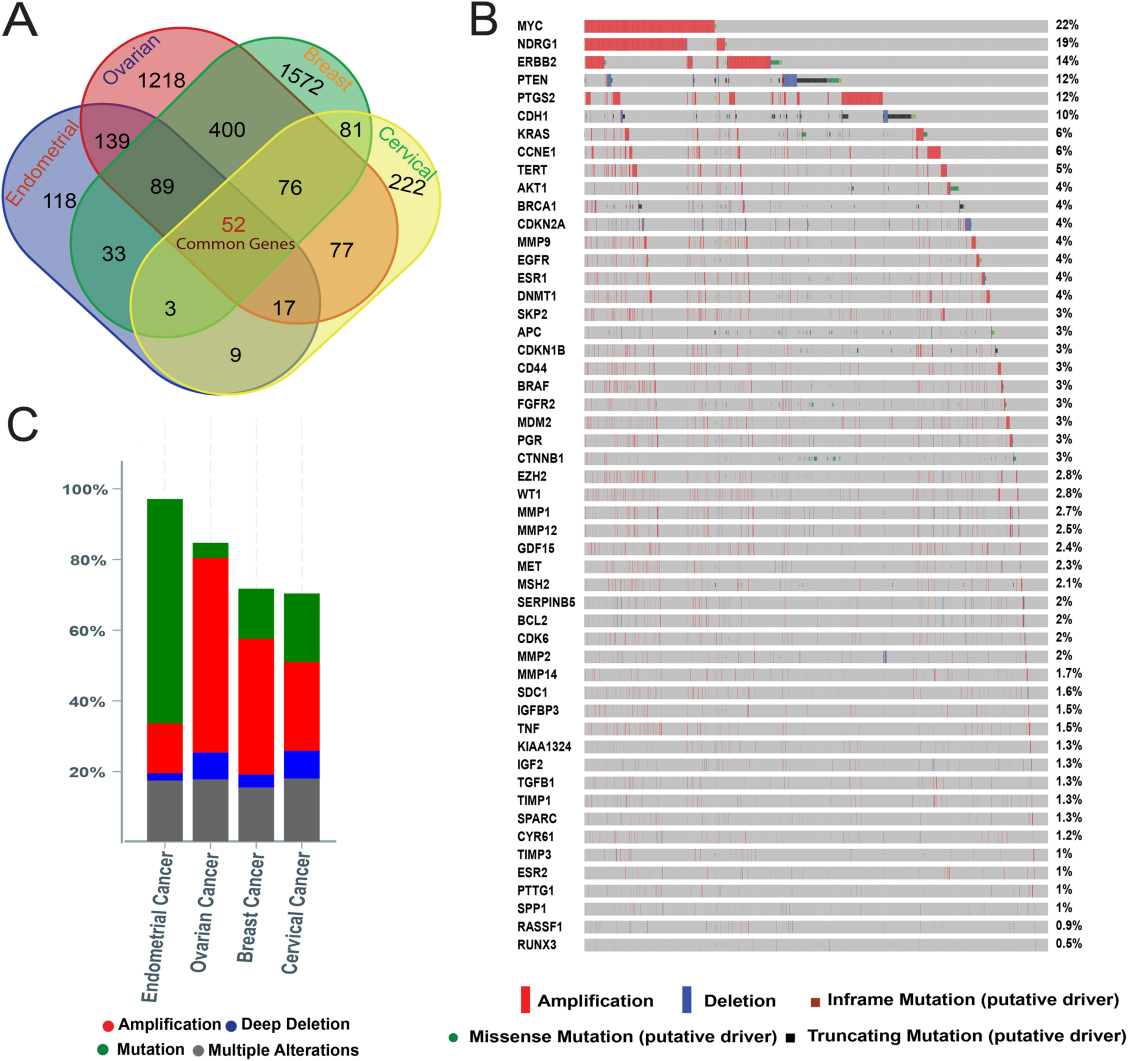
Overlapping and mutational analysis of four (Breast, ovarian, endometrial and cervical) cancer genes identified from four comprehensive literature-based databases. (A) Venn diagram showing number of exclusive and common genes implicated in breast, cervical, endometrial and ovarian cancers. (B) Mutational profile of the 52 common genes. (C) Graph showing cancer type and gene alteration frequency for the 52 common genes.

We conducted an integrated analysis of four cancers in women (breast, ovarian, endometrial and cervical) (Table S1) to identify the common genes implicated in cancer. Using a total of 460 endometrial, 2,068 ovarian, 2,308 breast and 537 cervical genes, we identified 52 genes that are common to all (Fig. 1A). A total of 128 (76 + 52) genes are common to breast, ovarian and cervical cancers; 141 (89 + 52) genes in breast, ovarian and endometrial cancers; 55 (3 + 52) genes in breast, cervical and endometrial cancers; 71 (17 + 52) genes in ovarian, endometrial and cervical cancers; 209 (81 + 76 + 52) genes in breast and cervical cancers; 174 (33 + 89 + 52) genes in breast and endometrial cancers; 528 (400 + 76 + 52) genes in breast and ovarian cancers; 205 (77 + 76 + 52) genes in ovarian and cervical cancers; 280 (139 + 89 + 52) genes in ovarian and endometrial cancers; and 64 (9 + 3 + 52) genes in endometrial and cervical cancers. A total of 1,572, 222, 118 and 1,218 genes were identified only for breast, cervical, endometrial and ovarian cancers, respectively. As cervical cancer is closely related to HPV infection, we additionally conducted a separate integrated analysis excluding cervical cancer. We also found that 141 genes in breast, ovarian and endometrial cancers (Fig. S2), which shows the same results we observed in four cancer analysis.

Systematic investigations of common driver gene sets among multiple cancers are useful for deciphering the molecular mechanisms and underlying pathways of cancer types. This study was, therefore, focused on the 52 identified genes common to breast, ovarian, endometrial and cervical cancers. Mutational analysis of these common cancer genes compares the frequency of genetic alteration. We conducted oncoprint analysis for the 52 genes (Table S2) to develop a concise and compact graphical summary of genomic alterations in multiple genes across 5,919 tumor samples (from 5,880 patients), which were extracted from TCGA datasets using cBioportal. There are many potential causal reasons for these mutations including genetic, cellular and environmental level. For example, aging is one of the major factors that could increase the mutational rate. For the TCGA breast cancer cohort (over 800 patients), the average diagnosed age was 60–65 years (Cerami et al., 2012).

From the oncoprint analysis, we identified that 4,401 patients (75%) had an alteration in at least one of the 52 genes. The highest mutation frequency was found in *MYC* (22%) followed by *NDRG1* (19%), *ERBB2* (14%), *PTEN* (13%), *PTGS2* (13%) and *CDH1* (11%) (Fig. 1B). For *MYC, NDRG1, ERBB2* and *PTGS2*, most of the alterations were amplifications and missense mutations. Out of the total 1,289 alterations in *MYC*, 0.30% are due to somatic mutations, and 0.35% have missense mutation. Alteration in *NDRG1* was observed in 1,109 samples, of which 0.50% have somatic mutation, 0.51% samples have missense mutation with unknown significance, and only 0.05% have truncating mutation. For *ERBB2*, 826 samples have alterations with somatic mutation in 2.6%, putative driver missense mutation in 2.89%, and truncating mutation with unknown significance in 0.05% samples. Similarly, *PTGS2*, which shows alteration in 737 of samples, where the somatic and missense mutations were 0.50% and 0.52%, respectively. In contrast, *PTEN* and *CDH1* showed different types of mutations. For *PTEN*, out of 745 altered samples, truncating mutation was the most common (7.16%) followed by putative driver missense mutation (4.92%) and deep deletion (4.5%). In *CDH1*, 631 samples were altered with greatest as somatic mutations (8.20%). *MMP14, MDM2, ERBB2, SKP2* and *PTGS2*, which were previously identified as OGs in human cancer (Gstaiger et al., 2001; Ling et al., 2017; Madson et al., 2006; Majumder et al., 2015; Zhang & Wang, 2000), also have higher rate of amplification in mutation data. Together with *PTEN* and *CDH1*, *CDKN2A* and *APC* showed a higher rate of deep deletion; all four are TSGs (Sonkin et al., 2013). Along with *ERBB2* and *PTEN*, a significant level of missense mutation was observed in *KRAS* (2.5%) and AKT1 (1.52%) (Table S3). In addition to point mutations, we also found that the copy number variants are common in our focused 52 genes. For *MYC*, 1,289 samples could be found in amplification events. Similarly, NDRG1 also have 19% amplification rate in 1,109 samples. These amplifications may imply their common oncogenic roles in the development of the four cancers in women. Overall, 20% of multiple alterations were found in each of breast, ovarian, endometrial and cervical cancers. Mutation rates were highest in endometrial cancers, but these showed the least deletion and amplification among the four cancers. The highest frequency of amplification was observed in ovarian cancer (Fig. 1C).

To explore the potential functional complementarity for the 52 genes, we conducted a mutual exclusivity analysis based on the mutational profile of all 6,366 tumor samples. The results showed that in most cases alteration of one gene function alters the function of another gene. In total 135 gene pairs were with mutually exclusive patterns, of which 22 pairs were significant (*P* < 0.05) and seven pairs highly significant (*P* < 0.001) (Table S4). For example, inactivation of *CD44* may also inactivate other genes like *CDKN2A, CDKN1B, CYR61, ESR1, EZH2, FGFR2, IGF2, KIAA1324, MDM2, MET, MMP1* (Table S5). Among the 22 significant (*P* < 0.05) gene pairs, *CDH1* showed mutually exclusivity with nine genes (*CCNE1, EGFR, GD15, MYC*, *NDRG1, SKP2, TERT, TGFB1 and TNF*), *AKT1* with four genes (*CTNNB1, MYC, NDRG1* and *PTEN*), *NDRG1* with four genes (*AKT1, CDH1, CTNNB1* and *PTEN*), *CTNNB1* with four genes (*AKT1, ERBB2, MYC* and *WT1*), and *PTEN* with a single gene (*NDRG1*).

**Figure 2:**
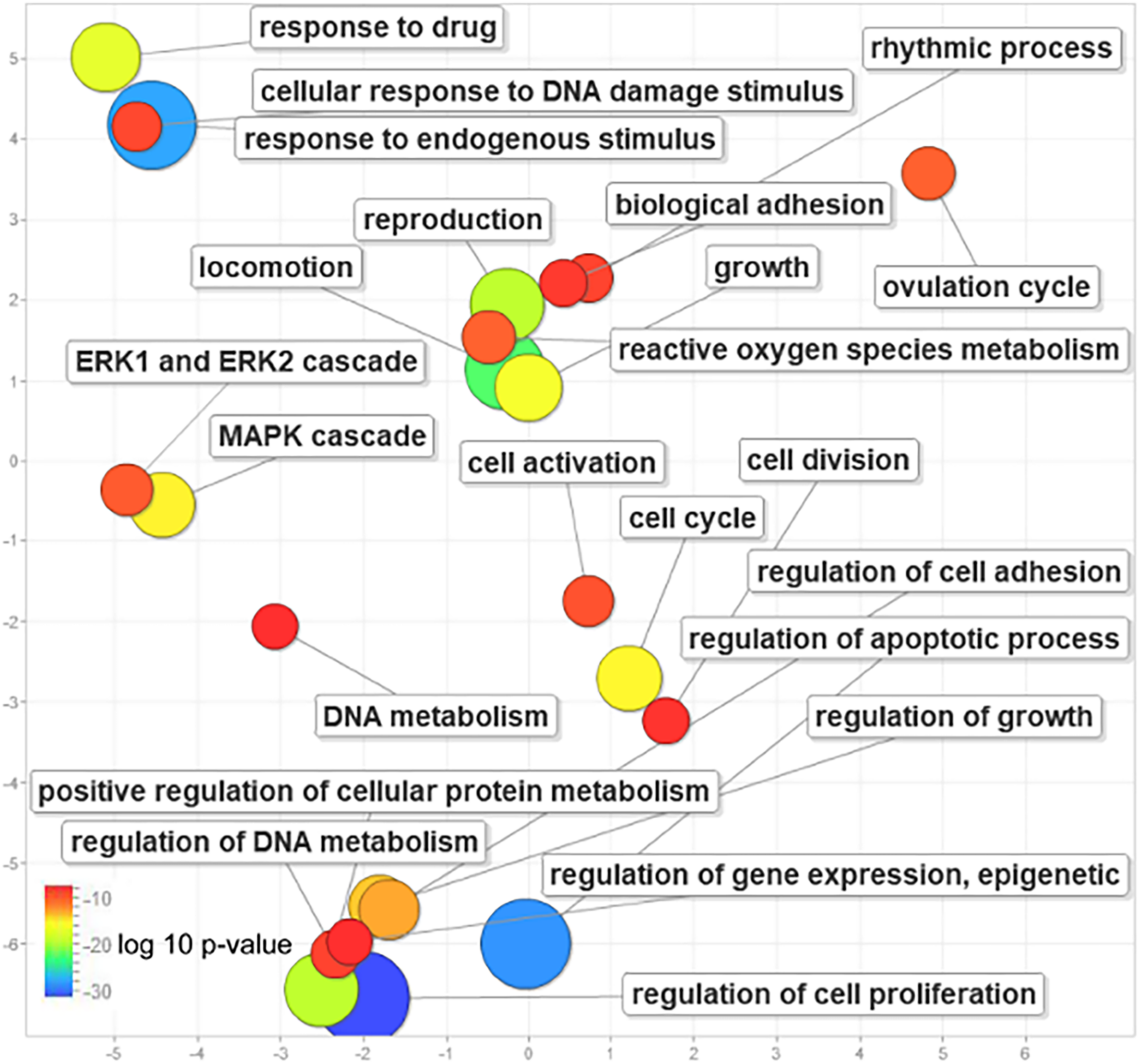
Visualization of functional enrichment analysis results showing the statistically (log10 *P*-value) over-represented gene ontology terms for 52 genes and their roles.

To investigate the general functions for the 52 genes, we conducted a functional enrichment analysis using Toppfun. Results revealed that these genes are enriched with growth (GO: 0040007 log10 *P*-value = −15.372), cell proliferation, cell death (GO: 0010941, log10 *P*-value= −28.4572), regulation of gene expression, epigenetic control, ovulation cycle, DNA metabolism, protein phosphorylation and signal transduction by phosphorylation (Fig. 2; Table S6). We found that ˜80% genes are active in regulating cell proliferation (GO: 0042127, log_10_ *P*-value = −31.2716). Interestingly, 38 genes were active in response to endogenous stimuli (GO: 0009719, log_10_ *P*-value = −27.6021) and nine genes (Table S6) were involved in the ovulation cycle (GO: 0042698, log_10_ *P*-value = −9.8386). These nine genes except *ERBB2*, response to endogenous stimuli and are involved in the ovulation cycle.

### Biological network integration for common genes

**Figure 3:**
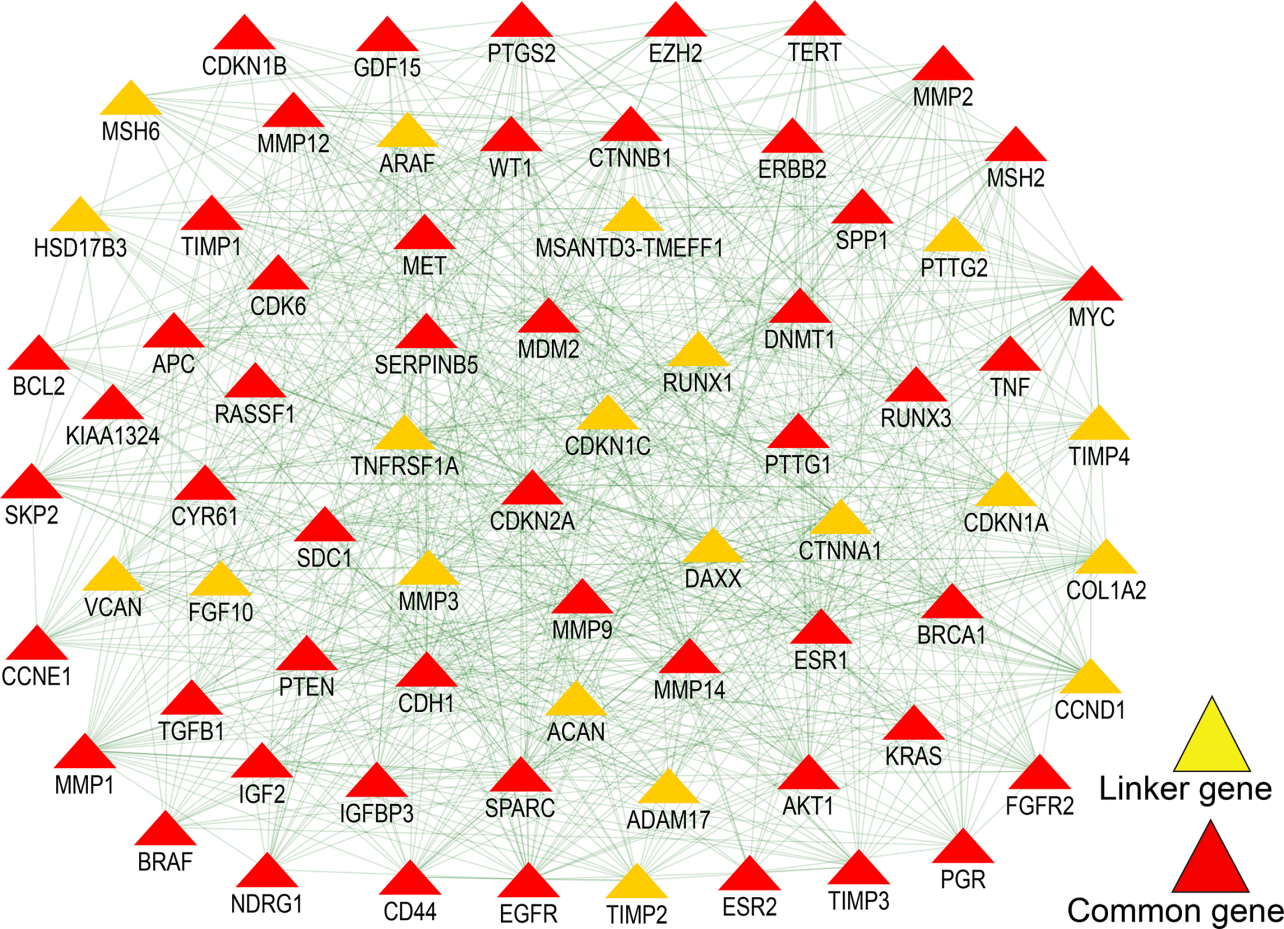
The network of the common 52 genes. The network represents the molecular function-based relationship between these 52 genes and the novel linker genes in cancer development. Red triangles represent four cancer (Breast, ovarian, endometrial and cervical) related genes and yellow triangles indicate linker genes.

**Figure 4:**
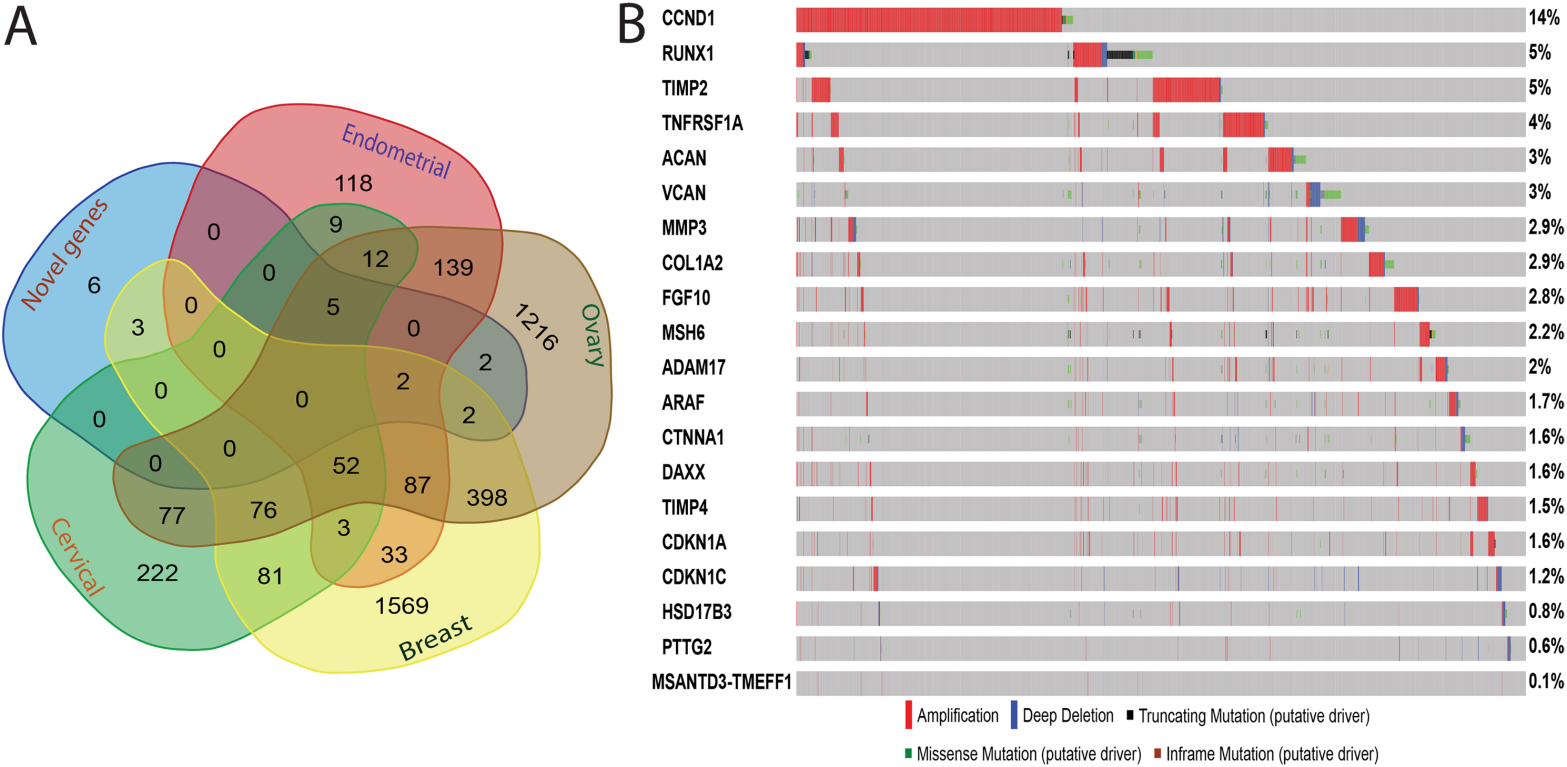
Identification of the role of 20 novel genes in four cancers in women, and their mutational analysis. (A) Venn diagram showing overlapping of novel genes, breast, ovarian, cervical and endometrial cancers genes. (B) Mutational profile for the 20 novel genes.

Network analysis showed that 12 genes (Table S7) are involved in fibroblast growth factor receptor signaling pathway. The epidermal growth factor receptor signaling pathway (FDR = 1.55e-8) and positive regulation of MAPK cascade also had 12 genes each. Regulation of the *ERK1* and *ERK2* cascade (FDR = 2.43e-6) network included eight genes (*TNF, CD44, CYR61, EGFR, ERBB2, FGFR2, FGF10, BRAF*). Additionally, we identified 20 novel driver genes (Fig. 3), which were not found in any of the four cancer databases. Interestingly, the overlapping analysis showed that only six of the genes are not involved in any of the four cancers (Fig. 4A). Two genes (*DAXX, ADAM17*) are involved in ovarian cancer and three genes (*TNFRSF1A, TIMP4, COL1A2*) are exclusive to breast cancer (Table S8). Two genes (*CTNNA1, CDKN1C*) are common in breast and ovarian cancers. Five genes (*MMP3, CDKN1A, RUNX1, TIMP2, CCND1*) are common in cervical, ovarian, endometrial cancers, but not in breast cancer. Two genes (*VCAN, MSH6*) are common in endometrial, breast and ovarian cancers. Mutational analysis of 20 novel genes (Fig. 4B) identified the highest mutational frequency in a cyclin D protein family gene, *CCND1* (14%), and the lowest in *MSANTD3-TMEFF1* (0.1%). All 20 genes showed some degree of amplification. Concordant amplification was found mostly in genes *TIMP4, TIMP2, MMP3, CCKN1A* and *CCND1*. Additionally, *MMP3* had deep deletion and missense mutation, *MSH6* had truncating and missense mutation, and *RUNX1* had deep deletion, missense and truncation mutations.

### Identification of endogenous stimulus genes playing a dual role in gynaecological cancers

Functional analysis of the 52 common genes identified that 38 play a role in response to endogenous stimuli (Table S6). Functional analysis of 20 novel genes showed that 12 genes are involved in endogenous stimulation (Table S9). We then analyzed the 38 genes for mutational frequency. In total, 5,949 samples were used for mutation profile analysis, of which 80% of the genes were mutated, including eight genes that were common in both endogenous pathways and ovulation cycle functions. For example, *CTNNB1*, an oncogene of many cancers, was found to be associated with endogenous stimulation, epigenetic control and ovulation. We had observed more than 150 mutations (3%) in *CTNNB1* (Fig. 1B), which is one of the main determining factors in cell division through regulating the transition from G_1_ to S phase. Two genes, *DNMT1* and *CTNNB1*, are known to have an epigenetic role in malignancy and were found to be endogenous stimulants.

### Network analysis of ovulation cycle related genes

**Figure 5:**
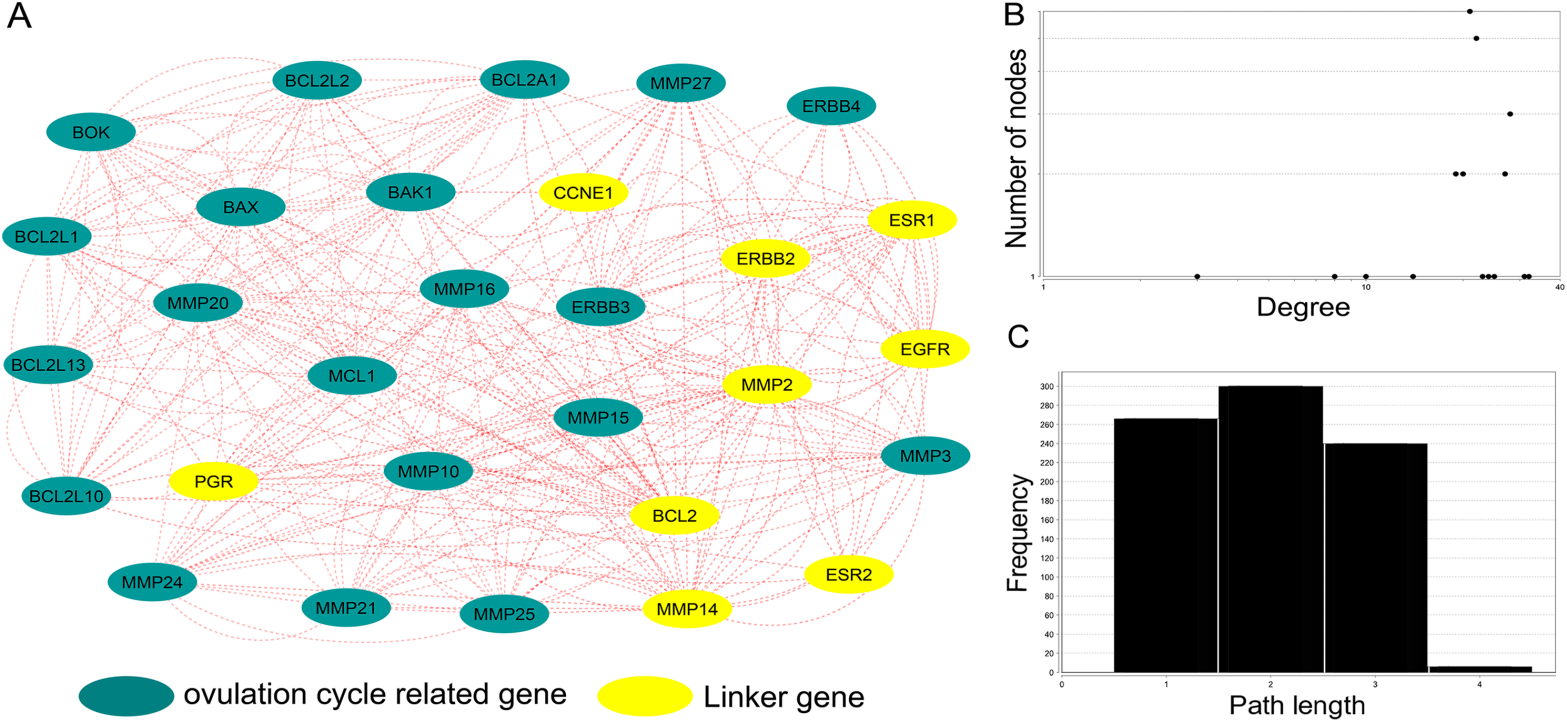
Network analysis for nine ovulation cycle genes common to four cancers (breast, ovarian, endometrial and cervical). Yellow circle presents ovulation cycle-related genes. (A) Visualization of network connectivity and different mapping path using Cytoscape. (B) Network Analyzer shows charts of the distribution of node degrees. (C) Shortest path lengths.

Among the 38 genes showing the response to endogenous stimuli, eight genes were associated with the ovulation cycle. We applied network analysis to identify the global connection and the functions of the ovulation cycle genes. Nine genes (*CCNE1, ERBB2, EGFR, ESR1, ESR2, BCL2, MMP2, MMP14, PGR*) of the 52 common genes were identified by functional enrichment analysis and were used in network analysis. From the derived network and focusing on genes with the highest number of interactions, we found 26 linker genes (Table S10), with *MMP2* the highest (21) and *ERBB2* the lowest connections (1). Among the other genes, three genes (*BAK1, BCL2A1* and *MMP27*) have 20 connections, two (*BCl2L13*, *MCL1*) have 19 connections, one (*ERBB3*) has 18 connections, two (*MMP21* and *MMP3*) have 16 connections, one (*MMP25*) has 15 connections, one (*MMP24*) has 13 connections, two (*BOK* and *MMP15*) have 12 connections, two (*EGFR* and *ESR1*) have 11 connections, four (*BAX, BCL2L10, ERBB4 and MMP2*) have 10 connections, one (*MMP10*) has eight connections, one (*MMP16*) has seven connections, one (*BCL2L1*) and six connections, three (*BCL2L2*, *MMP14* and *PGR1*) have two connections, and one (*ERBB2*) has only one connection. *EGFR, ERBB3, MMP20, MMP27, MCL1, BCL2A1* and *BAK1* appear to be important genes for cancer progression due to their frequent association with recurrent cancer-related genes in women (Fig. 5A). However, the ovulation cycle gene, *CCNE1* have no connection in the network. The degrees of the nodes in the map fit a power law distribution *y* = *ax^b^*, where *a* = 0.708, and *b* is an exponent with an estimated value of 0.272. The correlation between the given data point and the corresponding point on the fitted curve is 0.209 (*R*^2^ = 0.074) (Fig. 5B). Shortest path length distribution analysis shows that the average length of the shortest path is six (Fig. 5C).

## Discussion

### Common driver genes implicated in four cancers in women

Since the completion of human genome sequencing, a large number of gene mutations have been identified that can be associated with human cancers (Nik-Zainal et al., 2016). Computational prediction of driver genes involved in different types of cancers may lead to the development of measures for controlling cancers. Detection of functional driver pathways of common driver genes is necessary for personalized therapy and precision medicine in cancer treatment. This study identified common driver genes implicated in four cancers: breast, ovarian, cervix and endometrial cancer.

By mining four different cancer gene databases our study identified 52 genes common in all four cancer types. Mutational analysis provided evidence of the magnitude of genomic alteration of these genes. Similar to what has been shown previously (Nik-Zainal et al., 2016), the highest rate of mutation was observed in *MYC* (22%). In addition, the higher rate of mutation frequencies in previous studies indicated that genes *ERBB2, PTEN, PTGS2* and *CDH1* are involved in the regulation cancers in women (Kuusisto et al., 2011; Liao & Dickson, 2000; Revillion, Bonneterre & Peyrat, 1998; Smith, Kappler & Ethier, 2017). For the first time, we identified a higher rate of mutation in *NDRG1* (19%), which is a member of the N-myc down-regulated gene family, and belongs to the α/β hydrolase superfamily but does not have a hydrolytic catalytic site. *NDRG1* plays an active role in protection from ischemic cell damage (Lachat et al., 2002). *NDRG1* encoded protein is found universally in different tissues but primarily in the cytoplasm, cell membrane, and nucleus (Lachat et al., 2002). *NDRG1* is an iron-regulated gene (Kovacevic, Fu & Richardson, 2008) and is translated into cytoplasmic proteins involved in stress responses, hormone responses, cell growth and differentiation. All members of the *NDRG1* family are associated with various stages of differentiation from birth to adulthood concerned with cell proliferation, development and stress response (Ellen et al., 2008). However, depending on the cell type, *NDRG1* functions as both up-regulator and down-regulator. *NDRG1* suppresses metastasis of tumors in prostate, pancreatic, breast and colon cancers (Kovacevic, Fu & Richardson, 2008). In pancreatic cancer, *NDRG1* acts as an anti-tumor factor by repressing proliferation and impeding invasion and migration of the proteins *p-STAT3, PI3K, p-AKT, MMP2, MMP9* (Cen et al., 2017). It also upregulates the expression of *PTEN*, which is a tumor suppressor and downregulates the *PI3K/Akt* signaling pathways (Zhang & Yu, 2010). Kovacevic et al. (2016) demonstrated that *NDRG1* markedly decreased the expression and activation of *EGFR, HER2* and *HER3* proteins that are involved in activating a large number of downstream oncogenic signaling pathways. *NDRG1* also decreased the dimerization and *EGFR* activation in response to its ligand *EGF* and, consequently, decreasing phosphorylation and activation of the downstream *MAP2K* (a kinase enzyme, which phosphorylates mitogen-activated protein kinase). Ureshino et al. (2012) demonstrated that *NDRG1* plays its pivotal role in the malignant progression of gastric cancer through the epithelial-mesenchymal transition. Nagai et al. (2011) reported that *NDRG1* over-expression could play important roles in breast cancer progression and serve as useful biomarkers to improve breast cancer prognosis. However, the function of *NDRG1* in different cancers in women remains unclear.

We also conducted mutually exclusivity analysis to identify the functional mechanism of *NDRG1* in the oncogenesis and progression of the four cancers. Our study identified that *NDRG1* showed mutually exclusivity with *AKT1, CDH1, CTNNB1* and *PTEN*. Therefore, functional analysis of these genes provided evidence of the role and mechanism of *NDRG1* in gynaecological and breast cancers. Network analysis of 52 common genes identified 20 novel driver genes that are involved in the gene network systems but not identified as common to the four cancers in women. Out of 20 novel genes, *CCND1* (Cyclin D1) showed the highest mutational frequency (14%). Functional analysis revealed that *CCND1* regulates cell cycle progression (Matsushime et al., 1992) and is actively involved in tumor cell carcinogenesis specific to cell and tissue type (Carthon et al., 2005). Tumor cell proliferation is frequently associated with genetic or epigenetic alterations in key cell cycle molecules that regulate the activity of cyclin-dependent kinases (*CDK*s), which shows a dramatic periodicity in protein abundance throughout the cell cycle. For example, the subunits of *CDK4* and *CDK6* are activated in cell cycle G1/S transition (He et al., 2014). However, the two other genes, *RUNX1* and *TIMP2*, which are common in cervical, ovarian, and endometrial cancers, have 5% mutation for amplification and deletion. The *RUNX1* is a transcription factor that is well known as an essential regulator of diverse developmental processes including cell proliferation, apoptosis, development and cell lineage specification (Khawaled & Aqeilan, 2017). Further exploration of the functions of these 20 novel genes can help to identify their role in cancers in women.

### Genes causing four cancers in women play a dual role in endogenous hormonal regulation and ovulation cycle

Hormones are vital regulators of growth and development of the human body. Therefore, any endogenous hormonal imbalance influences cell division and multiplication and may trigger the development of cancer. Among the four cancers in women, three are hormone-sensitive: (i) breast cancer; (ii) ovarian cancer; and (iii) endometrial cancer (Dietel, Lewis & Shapiro, 2005). Over the years, epidemiological evidence has suggested that estrogen is a carcinogen and estrogen therapy may cause DNA replication error during cell division (Henderson & Feigelson, 2000). Errors in DNA replication ultimately gives rise to random mutations, creating malignant phenotypes, which continues throughout the cancer progression pathway (Henderson & Feigelson, 2000). Several endogenous stimulus genes are associated with these malignancies. In an attempt to identify these genes, we conducted a functional analysis of 52 common genes of cancers in women.

The apparent association of endogenous hormones in cancers in women, particularly breast and ovarian cancers, have been noted in several studies (Eliassen & Hankinson, 2008; Endogenous Hormones and Breast Cancer Collaborative Group, 2013). It was shown that sex steroid hormones (estrogen and testosterone) are linked to postmenopausal breast cancer risk in women. However, the underlying genetic control mechanism across multiple cancers in women is unclear. In this study, we explored multiple genetic control mechanisms related to endogenous hormonal regulation, and identified 38 genes involved in the endogenous stimulus pathways, that are common in the four cancers. Ovulation is triggered by hormones, and we identified that eight of the nine ovulation cycle genes are involved in hormone response pathways. For example, *CTNNB1* and *DNMT1*, which showed an epigenetic role in malignancy, were also found to be endogenous stimulants. As revealed in our study, *CTNNB1* showed a strong negative correlation with mRNA expression and methylation level in *BRCA* and *CESC* cell samples (Fig. S1). Fan et al. (2010) reported that *CTNNB1* facilitates follicle stimulation hormone-induced follicular growth and decreases follicle atresia (granulosa cell apoptosis) and represses luteinizing hormone (LH)-induced oocyte maturation, ovulation, luteinization and progesterone biosynthesis. It was also reported that *CTNNB1* prevents LH responses through reduced phosphorylation of cAMP-responsive element-binding protein and enhances follicle stimulating hormone (FSH) and LH actions in antral follicles (secondary ovarian follicle). Therefore, we suggest that *NDRG1* plays a dual role in cancers in women through endogenous stimulation and oviduct development.

A dual role in oncogenesis was also observed for other genes. For instance, the transforming growth factor beta 1 (*TGFB1*) is involved in endogenous stimulation and synthesis of LH (Ingman & Robertson, 2009) and causes the rupture of the mature ovarian follicle, resulting in egg release during ovulation. The mutation of insulin-like growth factor 1 (*IGF1*) gene delays egg development through the control of LH and this may result in ovulation failure (Baker et al., 1996). It is suggested that ovarian IGF-I expression serves to enhance granulosa cell FSH responsiveness by augmenting FSHR expression (Zhou et al., 1997). Previous studies identified that some genes can positively or negatively control cell proliferation and are thus active as both tumor suppressors and promoters. For example, *TGFB1*, which is the most abundant form of *TGFB*, has a dual function in cell malignancy. Firstly, it regulates the signaling networks of cell growth and differentiation by binding to the TGF-β receptor II (TGF-βRII) in the GS (glycine and serine-rich region) box (Massagué, Blain & Lo, 2000). Secondly, *TGFB1* actively decreases gastric cancer (Jin et al., 2007) but increases tumor progression in prostate cancer (Ewart-Toland et al., 2004). Allelic variants of the *TGFB1* gene were also demonstrated to increase the risk of breast cancer (Dunning et al., 2003; Hishida et al., 2003). Mutations in the alleles of the *TGFB* type II receptor are involved in ovarian cancers (Levy & Hill, 2006). The list of genes playing a dual role in four cancers in women can be utilized in cancer diagnosis, hence can increase the survival of women worldwide.

## Conclusions

This study investigated on four databases including ECGene, OCGene, G2SBC and CCDB to identify key driver genes involved in four cancers in women using overlapping analysis was implicated and 52 common genes were identified to be involved in four cancers. Mutational analysis evidenced the genomic alteration of key driver genes. Using network analysis, we identified 20 novel genes indirectly regulating these four cancers in women. Functional analysis of common driver genes along with 20 novel genes explains the genetic and physiological mechanism of oncogenesis causing these four cancers in women. Identification of genes involved in hormonal regulation in these four cancers in women provided a novel angle of deciding factors for cancer diagnosis and prognosis.

## Supplemental Information

10.7717/peerj.6872/supp-1 Supplemental Information 1 Differential methylation and expression. A) The correlation between DNA methylation and mRNA expression in the CTNNB1 gene of Breast Invasive Carcinoma. B) The correlation between DNA methylation and mRNA expression in the CTNNB1 gene of Cervical Squamous Cell Carcinoma. C) Differential methylation and expression in the DNMT1 gene of Breast Invasive Carcinoma. D) Differential methylation and expression in the DNMT1 gene of Uterine Corpus Endometrial Carcinoma. The expression has been plotted in fold change, log2 (X-axis) versus methylation rate presented as % (Y-axis) in CTNNB1 gene of breast invasive carcinoma (y=0.000015x+0.00730, stderr=0.035, corr=0.182) and cervical squamous cell carcinoma (y=0.000031x-0.03122, stderr=0.050, corr=0.300), and in DNMT1 gene of breast invasive carcinoma (y=0.000003x-0.00892, stderr=0.010, corr=0.120) and uterine corpus endometrial carcinoma (y=0.000002x-0.00807, stderr=0.005, corr=0.262).

10.7717/peerj.6872/supp-2 Supplemental Information 2 The overlapping of breast, endometrial and ovarian cancer related genes from databases. The venn diagram results represents the common genes between breast, endometrial and ovarian cancers. Genes were extracted from the four literature-based databases: EC gene, OC gene, G2SBC and CCDB.

10.7717/peerj.6872/supp-3 Supplemental Information 3 The gene list of endometrial, ovarian, breast and cervical cancers extracted from four literature-based databases.

10.7717/peerj.6872/supp-4 Supplemental Information 4 Gene symbols and full names of 52 common genes of four cancers collected from NCBI databases.

10.7717/peerj.6872/supp-5 Supplemental Information 5 The mutational frequency of 52 common genes. The online tool cBioportal was used to analyse the alteration frequency of 52 common genes using TCGA databases for breast, cervical, endometrial and ovarian cancers. The results were downloaded from the cBiportal tool after running the job. Finally, we sorted the downloaded data according to the genes alteration rate (%).

10.7717/peerj.6872/supp-6 Supplemental Information 6 Mutual exclusive alterations of 23 significant genes. Gene sets that are involved in top mutually exclusive patterns identified for the TCGA data.

10.7717/peerj.6872/supp-7 Supplemental Information 7 Mutual occurrence of 52 common genes. Summary statistics on mutual exclusivity and co-occurrence of genomic alterations in each pair of query genes. The P values are determined by a Fisher’s exact test with the null hypothesis that the frequency of occurrence of a pair of alterations in two genes is proportional to their uncorrelated occurrence in each gene.

10.7717/peerj.6872/supp-8 Supplemental Information 8 Functional analysis of 52 common genes. The functional analysis were conducted using the Toppfun online tool. The results provide the gene ontology term and the role of the genes.

10.7717/peerj.6872/supp-9 Supplemental Information 9 Network analysis of 52 common genes. The results of network analysis presented the pathways for each gene.

10.7717/peerj.6872/supp-10 Supplemental Information 10 Official names and functions of 20 novel genes.

10.7717/peerj.6872/supp-11 Supplemental Information 11 Functional analysis of 20 novel genes. The result of enrichment analysis showing the role of the genes and the gene ontology terms.

10.7717/peerj.6872/supp-12 Supplemental Information 12 Network analysis showing the number of connections of nine ovulation cycle genes to indicate the maximum and minimum number of connections.

## Additional Information and Declarations

### Data Availability

The following information was supplied regarding data availability: Data was collected from the ECGene (http://ecgene.bioinfo-minzhao.org/), OCGene (http://ocgene.bioinfo-minzhao.org/), G2SBC (https://omictools.com/g2sbc-tool), and CCDB (http://crdd.osdd.net/raghava/ccdb/stat.php) databases, and can be found in Table S1.
