# Supplementary figures and images for: Integrative analysis of common genes and driver mutations implicated in hormone stimulation for four cancers in women
Source: PeerJ. 2019 Jun 6;7:e6872. doi: 10.7717/peerj.6872 (PMC6556371; doi:10.7717/peerj.6872)

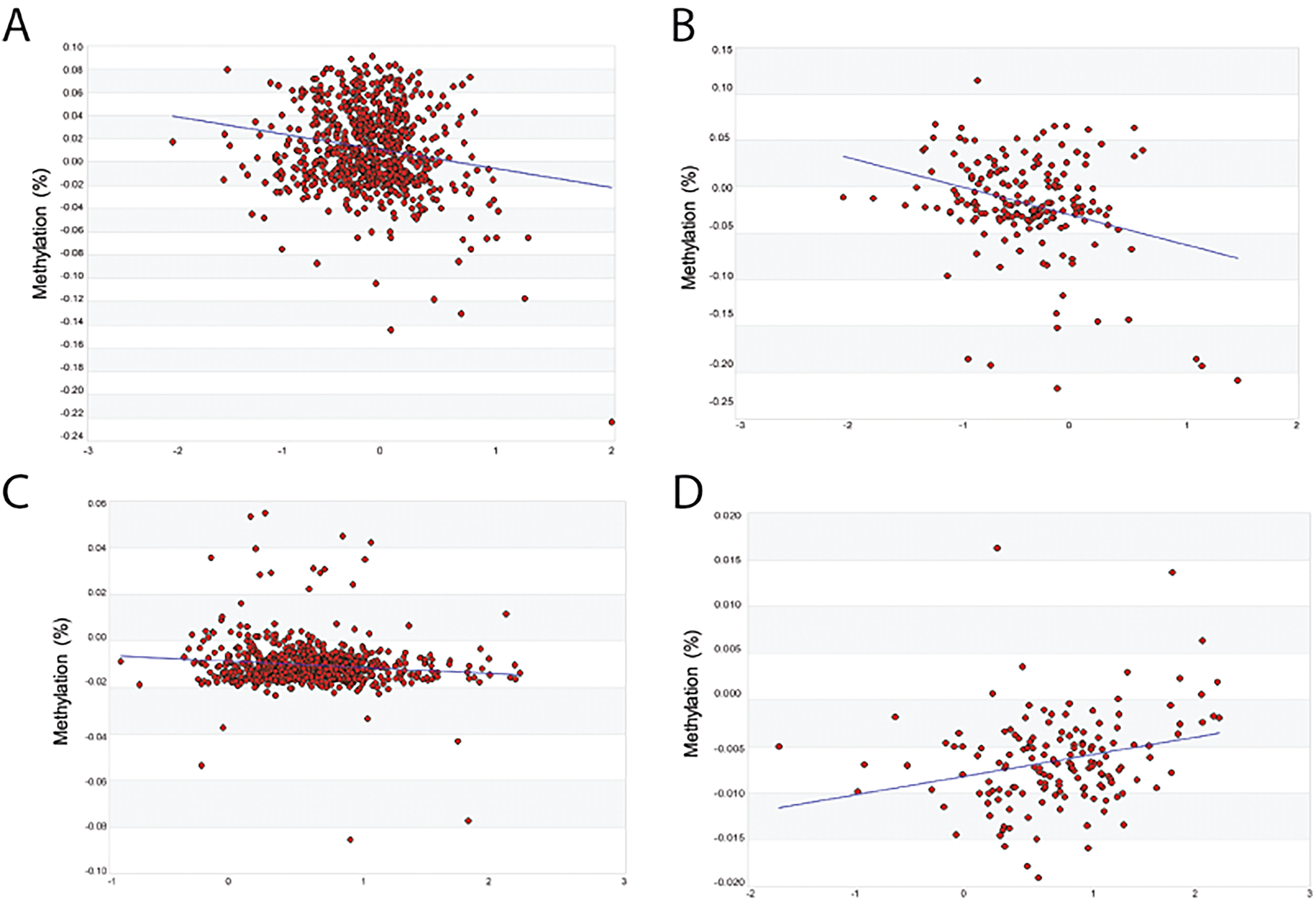

Supplement: Supplemental Information 1 — A) The correlation between DNA methylation and mRNA expression in the CTNNB1 gene of Breast Invasive Carcinoma. B) The correlation between DNA methylation and mRNA expression in the CTNNB1 gene of Cervical Squamous Cell Carcinoma. C) Differential methylation and expression in the DNMT1 gene of Breast Invasive Carcinoma. D) Differential methylation and expression in the DNMT1 gene of Uterine Corpus Endometrial Carcinoma. The expression has been plotted in fold change, log2 (X-axis) versus methylation rate presented as % (Y-axis) in CTNNB1 gene of breast invasive carcinoma (y=0.000015x+0.00730, stderr=0.035, corr=0.182) and cervical squamous cell carcinoma (y=0.000031x-0.03122, stderr=0.050, corr=0.300), and in DNMT1 gene of breast invasive carcinoma (y=0.000003x-0.00892, stderr=0.010, corr=0.120) and uterine corpus endometrial carcinoma (y=0.000002x-0.00807, stderr=0.005, corr=0.262). [file peerj-07-6872-s001.png]

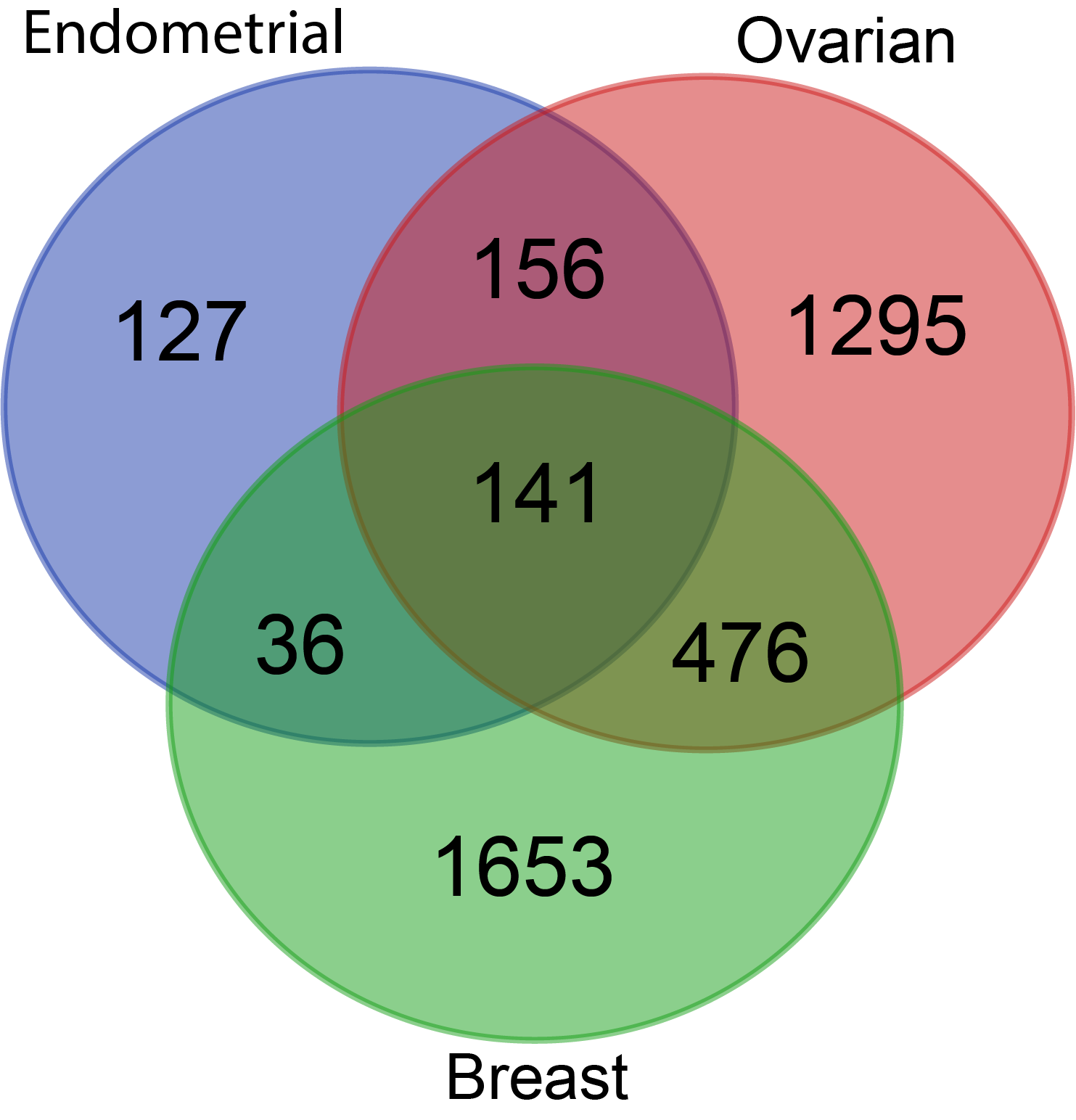

Supplement: Supplemental Information 2 — The venn diagram results represents the common genes between breast, endometrial and ovarian cancers. Genes were extracted from the four literature-based databases: EC gene, OC gene, G2SBC and CCDB. [file peerj-07-6872-s002.png]
